# Determining site occupancy of acetaminophen covalent binding to target proteins in vitro

**DOI:** 10.1002/ansa.202000182

**Published:** 2021-03-24

**Authors:** Timon Geib, Cristina Lento, Vanessa Marensi, Madhuranayaki Thulasingam, Jesper Z. Haeggström, Magnus Olsson, Derek J. Wilson, Elaine M. Leslie, Lekha Sleno

**Affiliations:** ^1^ Chemistry Department Université du Québec à Montréal Montréal Canada; ^2^ Department of Chemistry/The Centre for Research in Mass Spectrometry York University Toronto Canada; ^3^ Department of Physiology University of Alberta Edmonton Canada; ^4^ Division of Physiological Chemistry II Department of Medical Biochemistry and Biophysics Karolinska Institutet Stockholm Sweden; ^5^ Unit of Biochemical Toxicology Institute of Environmental Medicine Karolinska Institutet Stockholm Sweden

**Keywords:** acetaminophen, glutathione S‐transferase, multiple reaction monitoring, N‐acetyl p‐benzoquinone imine, protein covalent binding, protein modifications, reactive metabolite, site occupancy

## Abstract

Acetaminophen (APAP)‐related toxicity is caused by the formation of *N*‐acetyl *p*‐benzoquinone imine (NAPQI), a reactive metabolite able to covalently bind to protein thiols. A targeted liquid chromatography‐tandem mass spectrometry (LC‐MS/MS) method, using multiple reaction monitoring (MRM), was developed to measure APAP binding on selected target proteins, including glutathione *S*‐transferases (GSTs). In vitro incubations with CYP3A4 were performed to form APAP in the presence of different proteins, including four purified GST isozymes. A custom alkylation agent was used to prepare heavy labeled modified protein containing a structural isomer of APAP on all cysteine residues for isotope dilution. APAP incubations were spiked with heavy labeled protein, digested with either trypsin or pepsin, followed by peptide fractionation by HPLC prior to LC‐MRM analysis. Relative site occupancy on the protein‐level was used for comparing levels of modification of different sites in target proteins, after validation of protein and peptide‐level relative quantitation using human serum albumin as a model system. In total, seven modification sites were quantified, namely Cys115 and 174 in GSTM2, Cys15, 48 and 170 in GSTP1, and Cys50 in human MGST1 and rat MGST1. In addition, APAP site occupancies of three proteins from liver microsomes were also quantified by using heavily labeled microsomes spiked into APAP microsomal incubations. A novel approach employing an isotope‐labeled alkylation reagent was used to determine site occupancies on multiple protein thiols.

**Abbreviations**
ABC: ammonium bicarbonate buffer
APAP: acetaminophen
CAM: carbamidomethylated
CYP: cytochrome P450
d_4_‐HP‐IAM: *N*‐(4‐hydroxy[2,3,5,6‐d_4_]phenyl)‐2‐iodoacetamide
GST: glutathione *S*‐transferase
HP‐CAM: hydroxyphenyl‐carbamidomethylated
HP‐IAM: *N*‐(4‐hydroxyphenyl)‐2‐iodoacetamide
hSA: human serum albumin
IAM: iodoacetamide
MeOH: methanol
MRM: multiple reaction monitoring
NAPQI: *N*‐acetyl *p*‐benzoquinone imine
RLM: rat liver microsomes
RLS: rat liver S9 fractions
SO: site occupancy.

## 1 INTRODUCTION

Mass spectrometry is an extremely powerful tool for highly sensitive and selective detection of peptides and proteins in complex biological matrices. We can successfully identify and pinpoint sites of protein modifications, however, a real challenge still remains when it comes to the quantitation of these modifications even in relative terms to their unmodified counterparts. Protein modifications can occur from the covalent binding of reactive drug metabolites. For instance, reactive metabolite formation and liver protein binding of the common antipyretic and analgesic acetaminophen (APAP) have been attributed as the main cause of toxicity,^1^ leading to acute liver failure.^2^ Covalent binding to nucleophilic cysteine thiols occurs upon APAP's cytochrome P450 (CYP)‐catalyzed bioactivation to *N*‐acetyl *p*‐benzoquinone imine (NAPQI) in hepatocytes.^1^, ^3^, ^4^ Mitochondrial protein adducts are directly correlated to the initiation of hepatotoxicity.^5^, ^6^ Resulting cell death is triggered by NAPQI‐protein adducts formation, and subsequent mitochondrial dysfunction and fragmentation of DNA.^7^, ^8^ Certain amounts of NAPQI can be detoxified through conjugation with GSH, enzymatically controlled by different GST isoforms.^9^ However, through staggered or inadvertent APAP overuse, the non‐toxic conjugation pathways can be saturated and APAP‐protein binding in the mitochondria can thus increase to tissue‐damaging levels.^9^ In a previous study, we have observed representative protein binding of NAPQI formed via APAP metabolism *in vitro* to cysteines in recombinant human GST alpha 1, mu 1 and 2, and pi (hGSTA1, M1, M2, and P1). Two of these hGSTs (M2 and P1) were modified at multiple cysteine sites.^10^ However, in order to properly assess the relative level of binding between different modification sites, a method needed to be developed with adequate quantitative value.

Site occupancy (SO) is often used to measure the extent of protein modifications. Determining the extent of protein modification is vital to understand the resulting effects on protein function. Alkylation of cysteine residues was shown to reduce enzymatic activity of target proteins based on modification/protein ratio,^11^, ^12^ underlining the importance for quantitative protein alkylation studies. For instance, Nerland et al. observed loss of enzymatic function for rat GSTM1 after carbamidomethylation at Cys115, not at the theoretically more reactive Cys87.^13^

**FIGURE 1:**
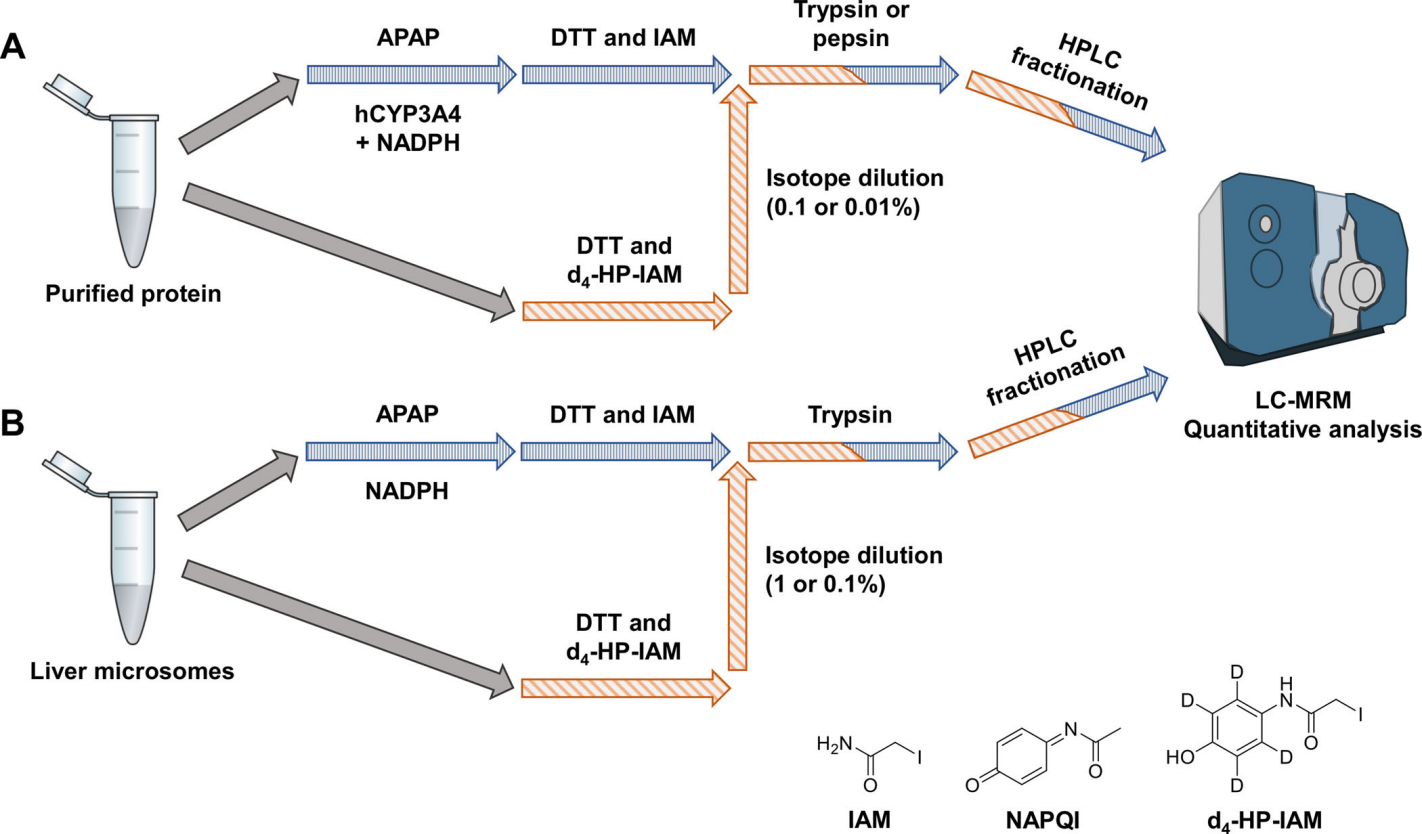
Scheme of analytical workflow. First, APAP was bioactivated using CYP3A4 Supersomes and combined with purified proteins (A) or using liver microsomes (B). Proteins were then reduced, alkylated, and digested with either trypsin or pepsin. Peptides were subjected to HPLC fractionation prior to LC‐MRM quantitation. Isotope dilution on the protein‐level was possible by alkylating purified proteins or microsomes using the custom d_4_‐HP‐CAM reagent. Heavily labeled proteins were spiked into the samples prior to the digestion step

APAP‐protein binding to several protein targets was quantified, including hGSTM2 and P1,^10^ as well as human and rat microsomal GST 1 (hMGST1 and rMGST1).^14^, ^15^ APAP was formed in vitro using hCYP3A4 Supersomes with the addition of individual target proteins (see Figure 1). APAP‐treated proteins were spiked with isotopically labeled protein. Following digestion, modified peptides were fractionated and subjected to targeted LC‐MRM analysis, using heavy peptides for normalization. Protein binding and corresponding SOs of purified proteins were also compared to APAP binding to hMGST1, and rMGST1 and rCYP2C6^15^ in human and rat microsomal incubations. These proteins and incubation systems were chosen to test the performance of the developed analytical workflow, which should be useful for future studies on specific mitochondrial protein binding in the context of APAP toxicity, for instance.^16^

## 2 MATERIALS AND METHODS

### 2.1 Materials

Human CYP3A4 Supersomes (with oxidoreductase and cytochrome *b_5_*, product #456202) and rat (Sprague‐Dawley) liver microsomes (RLM, product #452501) were from Corning (Corning, NY, USA). Mixed gender pooled human liver microsomes (HLM, product #452161) were from BD Gentest (Woburn, MA, USA). Standard peptides, LQQCPFEDHVKL and VFANPEDCAGFGK, were purchased from Biomatik (Cambridge, ON, Canada). Labeling agents *N*‐(4‐hydroxyphenyl)‐2‐iodoacetamide (HP‐IAM) and *N*‐(4‐hydroxy[2,3,5,6‐d_4_]phenyl)‐2‐iodoacetamide (d_4_‐HP‐IAM) were synthesized in‐house as previously described.^17^ Magnesium chloride and potassium phosphate (dibasic) were from Anachemia (Montréal, QC, Canada). Trypsin (TPCK‐treated, from bovine pancreas), pepsin (from porcine gastric mucosa), glucose‐6‐phosphate dehydrogenase (type XV, from baker's yeast), hSA, DTT, APAP, HPLC‐grade ACN and methanol (MeOH), iodoacetamide (IAM), and other chemicals were purchased from Sigma‐Aldrich (St. Louis, MO, USA). Ultrapure water was from a Millipore Synergy UV system (Billerica, MA, USA).

### 2.2 Purified protein preparation

Expression/extraction and purification of hGSTM2 and hGSTP1 (from *E. coli* and MCF‐7 cells, respectively),^10^ hMGST1,^18^ and rMGST1^19^ can be found in the Supporting Information.

### 2.3 Preparation of modified peptides, proteins, and microsomes

#### 2.3.1 Alkylated peptides

Standard peptide LQQCPFEDHVKL (20–30 nmol) in 100 mM ABC was reduced using DTT (250 nmol; 20 min at 37°C), and then alkylated by light or heavy (d_4_) hydroxyphenyl‐iodoacetamide to yield hydroxyphenyl‐carbamidomethylated (HP‐CAM) light and heavy‐labeled cysteines, or IAM (750 nmol; 45 min) at 37°C (in dark). Modified peptides were subjected to solid phase extraction (SPE) on OASIS HLB cartridges (1 cc (30 mg), Waters, Milford, MA). Methanol (1 mL) eluates were dried and reconstituted in 10% ACN prior to LC‐MS/MS. HP‐CAM and d_4_‐HP‐CAM‐peptides were used to evaluate results at different light/heavy ratios. Carbamidomethylated (CAM)‐peptides were used as an alternative internal standard. Light/heavy ratios were 1/1, 1/2.5, 1/5, 1/10, 1/25, 1/50, and 1/100 (each with three different constant light peptide/protein amounts of ∼0.2, ∼1.8, and ∼17.9 nmol on column).

#### 2.3.2 Alkylated proteins

Reductive alkylation of hSA, hGSTM2, hMGST1, or rMGST1 (1.8–4.2 nmol), or hGSTP1 (210–260 pmol) was performed in 100 mM ABC using DTT, and d_4_‐HP‐IAM (as above). Heavy d_4_‐HP‐CAM‐proteins were used as internal standards for isotope dilution experiments when determining SO of APAP modifications. Standard protein hSA were also alkylated with HP‐IAM, as well as IAM, to test assay accuracy at different analyte/internal standard ratios (same as above).

#### 2.3.3 Alkylated microsomes

RLM or HLM (200 µg each) were reduced and alkylated under the same conditions as above to yield d_4_‐HP‐CAM‐microsomes for isotope dilution.

### 2.4 APAP‐Protein binding

#### 2.4.1 APAP‐Protein binding via hCYP3A4 supersomes incubations

Incubation procedures were previously reported^10^ and optimal conditions were used. Briefly, hCYP3A4 Supersomes (130–200 µg total protein, 25 pmol hCYP3A4) and APAP (20 nmol) were incubated (37°C and 500 rpm) in the presence of a NADPH‐regenerating system, containing NADP^+^ (100 nmol), glucose 6‐phosphate (1 µmol), glucose‐6‐phosphate dehydrogenase (0.4 U), and magnesium chloride (1 µmol). Purified target protein (same amounts as above) in 100 mM phosphate buffer (pH 7.4) was added to react with produced APAP and incubated for 3 h, with a final volume of 200 µL.

#### 2.4.2 APAP‐protein binding to liver microsomes

APAP was incubated in RLM or HLM (200 µg each), respectively, as above. No other proteins were added as potential covalent targets.

### 2.5 Protein digestion

Following oxidative incubations with APAP, samples were diluted in ABC (100 mM), with 200 and 150 µL added for tryptic and peptic digestions, respectively. Protein samples were reduced and alkylated using DTT and IAM. Then, 25 µL of d_4_‐HP‐CAM protein or microsomes were added (in 100 mM ABC) to yield 1/100, 1/1000, or 1/10 000 d_4_‐HP‐CAM‐protein/starting protein ratios. All samples were digested with either 10 µg trypsin (4 h at 37°C), or 10 µg pepsin (following the addition of 400 µL solution of 1% formic acid in 10% methanol, 1 h at 37°C). Digests were cleaned‐up by SPE as described above.

### 2.6 2D‐LC‐MS/MS analysis

Dried extracts were reconstituted in 10% ACN and injected onto a ZORBAX Extend‐C18 column (250 × 4.6 mm, 5 µm; Agilent Technologies, Palo Alto, CA) on an Agilent 1200. High‐pH RP fractionation was performed with the same parameters as described previously.^10^ Fractions were concatenated, dried and reconstituted in 100 µL 10% ACN.

Relevant fractions were injected in triplicate (each 30 µL) onto an Aeris PEPTIDE XB‐C18 100 × 2.1 mm column, with solid core 1.7 µm particles (100 Å) fitted with a SecurityGuard ULTRA C18‐peptide guard column (Phenomenex, Torrance, CA) using a Nexera UHPLC system (Shimadzu, Columbia, MD). MRM was performed on a QTRAP 5500 (Sciex, Concord, ON) hybrid quadrupole‐linear ion trap system with a TurboIonSpray ion source in positive mode. The gradient (28 min) has previously been described.^10^ Individual MRM transitions (including hMGST1, rMGST1, and rCYP2C6) with instrumental parameters can be found in Supplementary Table S1 (Supporting Information). MRM transitions of hSA peptide (LQQCPFEDHVKL) were optimized from previous studies.^20^ Analyst software (Sciex, version 1.6) was used for data acquisition. Raw data were visualized with PeakView 2.2 and MasterView 1.1 (Sciex). MRM transitions were integrated using MultiQuant (Sciex, 3.0.3). One quantitative transition per peptide was chosen, however four transitions were monitored per peptide and ratios of transitions were verified for confirmatory purposes. Each quantitative transition was chosen based on highest S/N (minimum S/N of 10) with a higher fragment ion *m/z* than the precursor ion, and low intensity of isobaric interferences close to analyte retention.

## 3 RESULTS AND DISCUSSION

**FIGURE 2:**
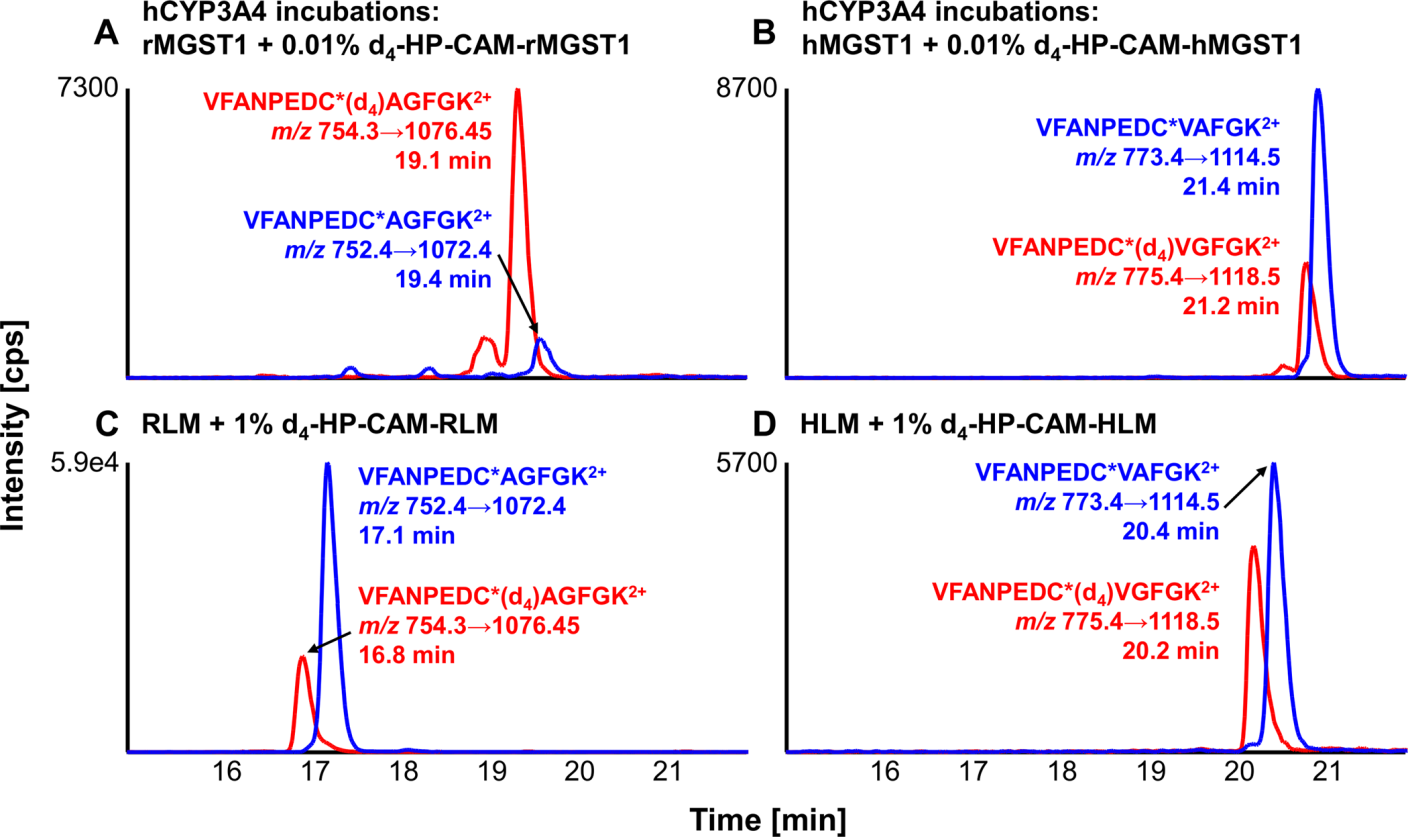
Representative chromatograms showing APAP‐modified peptides (VFANPEDC*AGFGK and VFANPEDC*VAFGK) from purified rMGST1 (fraction 3, A) and hMGST1 (fraction 4, B), and RLM (fraction 3, C) and HLM (fraction 4, D) samples. APAP‐modified proteins were spiked with 0.01% d_4_‐HP‐CAM‐modified MGST1s (A and B), and 1% d_4_‐HP‐CAM‐modified microsomes (C and D), followed by trypsin digestion, fractionation and LC‐MRM analysis. Light peptide MRM chromatograms are shown as blue traces and heavy peptides (*d_4_) as red traces

To quantify APAP‐related protein modifications, incubations were spiked with isotopically labeled internal standard (IS) proteins, which yielded the same peptides as their APAP‐modified counterparts, but contained heavy (d_4_) positional isomers of the modification. Following enzymatic digestion, APAP‐modified and IS peptides eluted very closely (see Figure 2), and showed identical MS/MS fragmentation behavior.^17^ Following an evaluation of this approach, appropriate amounts of spiked IS protein were chosen and these IS peptides were used for estimating APAP site occupancies on specific thiols in target proteins. Appropriate amounts of IS were important for adequate relative normalization due to varying ionization conditions and retention time variation caused from differing sample complexities. This can be seen in chromatograms of VFANPEDC*AGFGK peptide from rMGST1 digest as purified protein (Figure 2A) and in RLM (Figure 2C), as well as VFANPEDC*VGFGK peptide from hMGST1 digested from purified protein and in HLM (see Figure 2B,D).

### 3.1 Assay performance for relative quantitation

The ability to quantify protein modification sites was first evaluated using light (HP‐CAM) and heavy (d_4_‐HP‐CAM) modified peptide. Assay linearity was determined for light and heavy alkylated peptides and proteins at different isotope dilution ratios. A synthetic peptide containing a known APAP modification site, from hSA, were alkylated and compared to the alkylated protein, following by digestion via pepsin. The target peptic peptide was LQQCPFEDHVKL, containing the active site Cys34.

**FIGURE 3:**
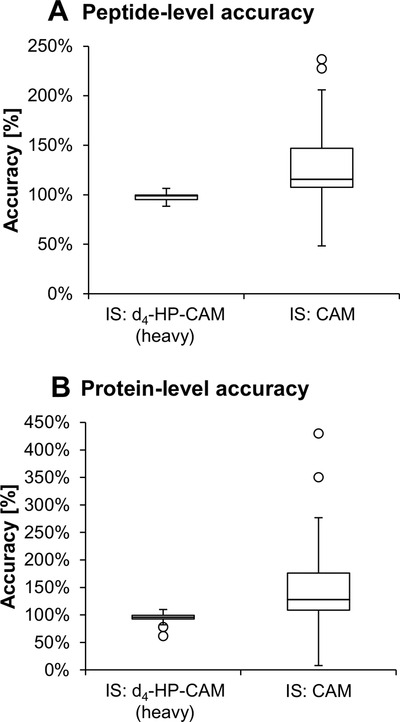
Accuracies of MRM area ratios (analyte/IS) of HP‐CAM‐modified (A) LQQCPFEDHVKL and HP‐CAM‐modified (B) hSA at seven different analyte/IS ratios (from 1/1 to 1/100) with three different, constant amounts of HP‐CAM‐peptide/protein. Box plots are shown to compare (A) peptide‐level and (B) protein‐level accuracies (closeness of area ratio to concentration ratio) using either d_4_‐HP‐CAM or CAM modifications as IS

Assay accuracy was assessed at seven different light/heavy ratios, with the amount of light HP‐CAM‐peptide being held constant at three different levels (Figure 3A). The same experiment was repeated on the protein‐level (hSA, Figure 3B). Using HP‐CAM‐modified peptide (or protein), at different light to heavy ratios, was compared to CAM‐peptide as internal standard (Figure 3). Median accuracies standards were 98.7% and 95.7% using d_4_‐HP‐CAM‐labeled peptide and protein, respectively. Performing relative quantitation with CAM‐peptides and proteins at identical light peptide/CAM‐peptide ratios led to similar median accuracies, 115.5% and 127.8%, on the peptide and protein‐level, respectively. However, standard deviations were smaller with an interquartile range (IQR) of 4.5% and 7.0% for d_4_‐HP‐CAM peptide and protein IS, respectively, compared to IQRs of 39.4% and 67.2% for CAM‐peptide IS and CAM‐protein IS, respectively (see Figure 3C,D). Using isotope dilution at a light/heavy ratio close to 1:1 was selected for most reliable relative quantitation. Results also confirm that using a light and heavy isotope pair yields much better accuracies than using the same peptide with a different chemical modification. The latter approach is what has been employed most often for quantifying site occupancies in previous reports,^21^, ^22^, ^23^ for instance using the ratio of modified to unmodified peptide in the same sample. However, the modified‐to‐unmodified peptide ratio can be quite small in many cases, making the reliability of such an approach questionable. Also depending on the modification, a very different chromatographic retention, ionization efficiency and fragmentation behavior can amount to extreme differences in signal intensity even for the case of an equimolar mixture of modified‐to‐unmodified peptide.

### 3.2 Relative quantitation of APAP site occupancy of target proteins

Relative occupancy quantitation of APAP modification of target proteins at multiple possible sites was achieved by isotope dilution prior to proteolysis. SO was calculated as shown in Equation 1, with integrated peak areas (A) of modified peptides at different isotope dilutions (i.d.) (1%, 0.1%, or 0.01%):

(1) $$SO=\frac{A(NAPQI-peptide)}{A(d_{4}-HP-CAM-peptide)}i.d.\%$$

Human GST P1, obtained using two different expression strategies, was investigated for differences in APAP binding. Wild‐type and His‐tagged hGSTP1 were used to compare the effect of purifying GSTs using a GSH affinity‐based clean‐up step or affinity purification on a nickel‐based column, respectively. The wild‐type GST stock solution had the potential for residual free GSH in the stock solution, as well as the possibility for glutathionylated thiol sites within the protein which would block APAP binding.^12^

**FIGURE 4:**
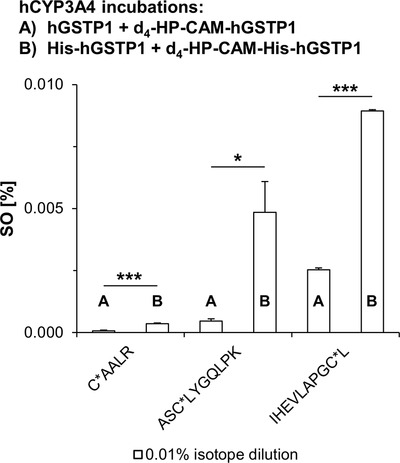
Quantitative differences in SOs of MCF‐7‐hGSTP1 and MCF‐7‐His‐hGSTP1 were determined using APAP bioactivation via hCYP3A4 Supersomes and 0.01% isotope dilution. SD from repeated injections (*n* = 3) is shown as error bars. Statistically significant difference between SOs of both protein variants was determined by two‐tailed two‐sample *t*‐test, assuming unequal variances (*P ≤ .05, ***P ≤ .001)

A direct comparison of the SO of three MCF‐7‐hGSTP1 peptides (using 0.01% isotope dilution) showed 3 to 10x higher alkylation of GSH‐free His‐tag MCF‐7‐hGSTP1 (see Figure 4). Differences in APAP modification of C*AALR, ASC*LYGQLPK and IHEVLAPGC*L peptides were found to be statistically significant with two‐tailed *p*‐values of 0.020, < 0.001, and 0.026, respectively. The absence of nucleophilic GSH during the purification process allowed for higher APAP binding potential with equal quantities of protein.

**FIGURE 5:**
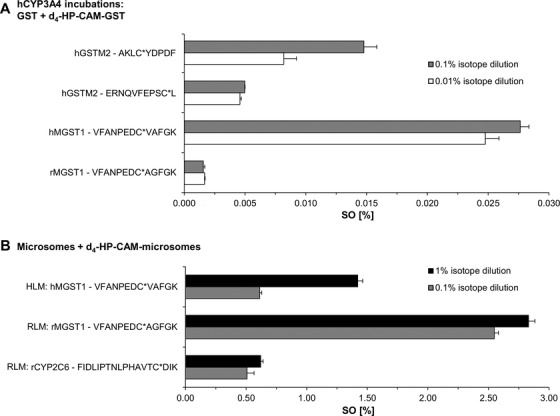
APAP site occupancies were calculated for GSTs spiked into hCYP3A4 Supersomes incubations using 0.1% and 0.01% isotope dilution (A). APAP‐modified hMGST1, rMGST1 and rCYP2C6 in human or rat liver microsomes were also quantified using d_4_‐HP‐CAM‐modified microsomes (at 1% and 0.1% isotope dilution) (B). Standard deviations from repeated injections (*n* = 3) are shown as error bars

APAP SO was also determined for three other purified GST proteins (hGSTM2, hMGST1, and rMGST1) as well as three proteins found in liver microsomes using the same workflow with isotope dilution prior to digestion (Figure 1). Calculated SO of all cysteine sites can be found in Table S2. A comparison of purified GST SOs and microsomal protein SOs can be found in Figures 5A and 5B, respectively. Relative SOs were considerably lower in incubations using purified proteins and hCYP3A4 Supersomes, compared to microsomal incubations. This could be a consequence of the process by which the reactive metabolite was formed as well as the proximity of the modified microsomal proteins (MGST1 and CYP2C6) to the reactive NAPQI as it was being formed. Briefly, combining SO results from two different isotope dilution strategies (0.1% and 0.01%), we observed a total SO range from 0.0016% to 0.0276% in purified GSTs. SOs of rat and human MGSTs and rCYP2C6, contained in human and rat liver microsomes used for the metabolic transformation of APAP to APAP in *in vitro* incubations, ranged from 0.508% to 2.831%. Differences in SOs of incubations using hCYP3A4 and incubations using microsomal liver fractions could hint at differences of oxidative capabilities of these incubation systems forming NAPQI, or could be a result of the large quantitative and functional differences of starting target protein amounts (purified spiked‐in protein compared to protein in the microsomal mixture). Comparing SO of rMGST1 in RLM incubations and hMGST1 in HLM, SO on rMGST1 is 2.6× higher than on hMGST1 in HLM incubations (averaged 1% and 0.1% isotope dilutions, see Figure 5B). Rat microsomes are known to have higher CYP activity, and thus NAPQI formation. This could explain the reversed species SOs between purified rat and human MGST1s and protein from microsomes (see Figure 2). In incubations using purified MGST1s (see Figure 5A), SO on rMGST1 is 15.4× lower than on hMGST1 (averaged 0.1% and 0.01% isotope dilutions). In addition, we observed relatively high differences of SOs between different isotope dilutions for peptide AKLC*YDPDF of hGSTM2 (Figure 5A) and VFANPEDC*VAFGK in hMGST1 (Figure 5B). These variations might be cause by relatively low S/N of analyte or IS. For AKLC*YDPDF, average analyte S/N was 70 for all isotope dilution experiments. This is in contrast with an average S/N of 930 for peptide ERNQVFEPSC*L of the same protein, which yields higher reproducibility of SO quantifications. For the peptide VFANPEDC*VAFGK from HLM, IS signal of 0.1% isotope dilution was relatively low with an average S/N of 149.7, compared to an average S/N of 1206.3 in 1% isotope dilution runs. Thus, accuracy of SO quantification of VFANPEDC*VAFGK is therefore likely higher using 1% isotope dilution.

We were able to quantify eight APAP‐modified cysteine sites in purified GSTs, namely Cys115 and 174 (hGSTM2), Cys15, 48 and 170 (hGSTP1), and Cys50 (hMGST1 and rMGST1. Moreover, multiple cysteines in hGSTM2 and P1 were modified and allowed ranking of APAP affinities at different sites within the same protein. In hGSTM2, Cys115 showed a higher affinity to APAP than Cys174. The extent of modification on Cys115 is important to quantify since cysteine mutant studies previously presumed that Cys115 was involved in enzyme activity,^24^ based on the adjacent active site Tyr116.^25^ We observed that the SO on Cys115 on average 2.4x that on Cys174. Furthermore, comparing the three modification sites in (wild‐type) hGSTP1, we observed 40.0x and 5.5x higher SO of Cys170, respectively, for Cys15 and 48. Cys15 is in direct proximity to the GSH binding site Arg14.^26^, ^27^, ^28^, ^29^, ^30^, ^31^ Binding to Cys15 might thus affect GSH binding during catalysis. Cys48‐related binding is known to cause protein inhibition, also based on its proximity to GSH binding sites,^32^, ^33^ with subsequent disruption of the hGSTP1‐1 dimer structure.^12^ Also based on dimerization capabilities,^34^ modification on Cys170 is believed to influence enzyme function.^35^

The goal of this study was to introduce a tool for accessing APAP affinity, complementing previous binding studies, where modification sites were identified. For instance, a technique to analyze binding reactions in microsomal fractions from different species would be useful to compare site occupancy of several modified proteins that were previously identified (see Golizeh *et al*, and Shin *et al*).^14^, ^15^ This would allow for the study of APAP binding reactions in more complex environments, where binding competition between proteins might be present.

## 4 CONCLUSION

A quantitative investigation of APAP covalent binding to multiple proteins was performed and demonstrated the challenges in quantifying modified proteins and peptides. A heavy‐labeled alkylation reagent was used to prepare an appropriate standard for quantitation of site occupancies, while taking into account losses during digestion and other sample preparation steps. This method can be easily applied to APAP binding targets found in bottom‐up proteomics workflow. Through simple addition of a quantitatively heavy‐labeled protein or complex protein mixtures spiked‐in prior to digestion, this technique can complement standard proteomics workflow without extensively changing existing protocols and sample preparation. This tool proved most useful as a relative quantitation tool, comparing multiple sites/proteins in the same given sample (type), independent of its complexity. Additionally, comparing peptide‐level and protein‐level quantitation of modification sites showed the crucial importance of using protein‐level standards whenever possible for estimating site occupancy.

## CONFLICT OF INTEREST

The authors declare no conflict of interest.

## Supporting information

Supporting information

## Data Availability

The data that support the findings of this study are available in supplementary information provided and further information or data are available from the corresponding author upon reasonable request.
