# Supplementary material for: Determining site occupancy of acetaminophen covalent binding to target proteins in vitro
Source: Anal Sci Adv. 2021 Mar 24;2(5-6):263–71. doi: 10.1002/ansa.202000182 (PMC10989598; doi:10.1002/ansa.202000182)
Supplement: Supplementary file 1 — Supporting information [file ANSA-2-263-s001.pdf]

Supporting Information

Determining site occupancy of acetaminophen covalent binding to target proteins *in vitro*

Timon Geib,[1] Cristina Lento,[2] Vanessa Marensi,[3] Madhuranayaki Thulasingam,[4] Jesper Z. Haeggström,[4] Magnus Olsson,[5] Derek J. Wilson,[2] Elaine M. Leslie,[3] and Lekha Sleno[*,1]*

[1]Department of Chemistry, Université du Québec à Montréal, Montréal, Canada

[2]Department of Chemistry/The Centre for Research in Mass Spectrometry, York University, Toronto, Canada

[3]Department of Physiology, University of Alberta, Edmonton, Canada

[4]Division of Physiological Chemistry II, Department of Medical Biochemistry and Biophysics, Karolinska Institutet, Stockholm, Sweden

[5]Unit of Biochemical Toxicology, Institute of Environmental Medicine, Karolinska Institutet, Stockholm, Sweden

Corresponding Author:

*Prof. L. Sleno: P.O. Box 8888, Downtown Station, Montréal, Québec H3C3P8, Canada. Fax: +1-514-987-4054. Email: sleno.lekha@uqam.ca

Table of Content:

1. Supporting Information: Materials and Methods 2

1.1. Recombinant Expression or Extraction of GSTs 2

1.1.1. Recombinant Expression of hGSTA1, M1, M2 and P1 2

1.1.2. Recombinant Expression of hMGST1 3

1.1.3. Extraction of rMGST1 4

2. Supporting Information: References 7

3. Supporting Information: Tables 9

3.1. Table S1 9

3.2. Table S2 12

1. Supporting Information: Materials and Methods

1.1. Recombinant Expression or Extraction of GSTs

1.1.1. Recombinant Expression of hGSTM2 and hGSTP1

Recombinant hGSTM2 was expressed in *E. coli* (from laboratory stocks), as previously published.[1] Proteins were expressed using a modification of published protocols.[2,3] Overnight induction was proceeded when the culture reached OD_{600} = 0.6. Cells were lysed as previously described.[2] Protein concentration was determined with the Bradford assay, using bovine serum albumin as reference standard.[4] GST affinity purification was performed on a GSH-agarose column.[3] Purified hGSTM2 were dialyzed to remove GSH by 3 L changes against 500 μL into ammonium acetate for 48 h.

Two sets of hGSTP1s, hGSTP1 (wild type) and His-tag hGSTP1, were expressed in MCF-7 cells (American Type Culture Collection; Manassa, VA). His-tag hGSTP1 containing six histidine residues at its N-terminus was generated using the Agilent QuikChange Lightning site-directed mutagenesis kit (Mississauga, ON), using pcDNA3.1(+)-hGSTP1[5] as the PCR template according to the manufacturer's instructions. The mutagenic primers were from Integrated DNA Technology (Coralville, IA). The sequence of the forward primer was 5'…AAAAAGGATCCACCATGGCT<u>CATCATCATCATCATCAT</u>CCGCCGTATACCGTGG …3' (the codons for the six histidine residues underlined) and the reverse primer 5'…CGGCCCTCTAGACTCGAGTGACTGTTTCCCGTTGCCATTGATGG…3'. The entire hGSTP1 cDNA and His-tag was sequenced to confirm that the His-tag was incorporated and to ensure no unintentional mutations were incorporated during the PCR (Molecular Biology Servicing Unit, University of Alberta, Edmonton, Canada).

MCF-7 cells were maintained in DMEM supplemented with 4 mM L-glutamine, 1 mM sodium pyruvate, and 10% FBS. Cultures were maintained in a humidified incubator with 95% air/5% carbon dioxide and routinely tested for mycoplasma contamination (Universal Mycoplasma Testing Kit, ATCC). Generation of the MCF-7-hGSTP1 stable cell line has been described previously.[5] The MCF-7-His-hGSTP1 was generated in a similar manner except it was not cloned by limiting dilution. The MCF-7-hGSTP1 and MCF-7-His-hGSTP1 were maintained in the same media as the parental MCF-7 with the addition of 600 μg/mL of G418 (Geneticin).

MCF-7-hGSTP1 (2×10^7 cells) were re-suspended in 3 mL of Tris-sucrose-buffer (50 mM Tris, pH 7.4, 250 mM sucrose) containing calcium chloride (250 μM) and EDTA-free cOmplete™ protease inhibitors, and disrupted by nitrogen cavitation as described previously.[6] Cell homogenates were centrifuged at 500 g at 4°C for 10 min to remove nuclei and unbroken cells. Supernatants were then spun at 100,000 g at 4°C for 30 min to remove cellular membranes. The supernatant (cytosolic fraction) was retained and hGSTP1 purified using Pierce GSH-agarose using the batch method described in the manufacturer's protocol (Pierce Biotechnology Thermo Scientific, Rockford, IL). Then, hGSTP1 was eluted in 50 mM Tris, 20 mM GSH (pH 8) then diluted one-to-one with glycerol and stored at −80°C.

MCF-7-hGSTP1 (2×10^7 cells) were re-suspended in 3 mL of lysis buffer (300 mM sodium chloride, 25 mM sucrose, 20 mM imidazole, 0.1% CHAPS (*w/v*), in PBS at a final pH of 7.5) and incubated 10 min on ice. The suspension was then centrifuged at 16,000 g at 4°C for 10 min, supernatant collected, CHAPS added to 0.5% then incubated on ice for 10 min. A bicinchoninic acid protein assay was then performed, and the protein diluted to 1 mg/mL in lysis buffer with 0.5% CHAPS. His-hGSTP1 was then purified according to the QIAexpressionist protocol (5th edition, QIAGEN) using Ni-nitrilotriacetic acid batch purification. Ni-

nitrilotriacetic acid bound to His-hGSTP1 was washed with CHAPS-free lysis buffer and eluted in CHAPS-free lysis buffer containing 250 mM imidazole. His-hGSTP1 was then diluted one-to-one with glycerol and stored at −80°C.

1.1.2. Recombinant Expression of hMGST1

Human *MGST1* gene was cloned in pPICZA vector (Invitrogen, Carlsbad, CA) and expressed in yeast *Pichia pastoris*. The expression and solubilization conditions were essentially as described for MGST2.[7] The solubilized protein was passed through Ni Sepharose 6 Fast Flow column (GE Healthcare, Chicago, IL). The column was washed with five column volumes of buffer containing 25 mM Tris-hydrochloride (pH 7.8), 500 mM sodium chloride, 10% glycerol, 5 mM 2-mercaptoethanol, 0.05% *n*-dodecyl β-D-maltoside (DDM), 0.1 mM GSH and 20 mM imidazole followed by three column volumes of same buffer having 40 mM imidazole instead of 20 mM concentration and hMGST1 protein was finally eluted in buffer containing 300 mM imidazole. The peak fractions were pooled and passed through *S*-hexylglutathione agarose column (Abcam, Cambridge, United Kingdom; and GE Healthcare) after equilibration. The column was washed with three column volumes of buffer containing 25 mM Tris-hydrochloride (pH 8.0), 500 mM sodium chloride, 10% glycerol, 5 mM 2-mercaptoethanol, 0.05% DDM and 0.1 mM GSH. Protein was eluted with the same buffer containing 30 mM probenecid but without sodium chloride. The eluate was concentrated using Amicon Ultra-15 30 kDa centrifugal filter device (MilliporeSigma, Burlington, MA) and purified further by size exclusion chromatography using HiLoad 16/600 Superdex 200 pg (GE Healthcare) column. The final buffer used for size exclusion chromatography contained 25 mM Tris-hydrochloride (pH 8.0), 100 mM sodium chloride, 10% glycerol, 0.1 mM tris(2-carboxyethyl)phosphine, 0.03% DDM and 0.1 mM GSH.

The final fractions containing pure hMGST1 were concentrated to 32 mg/mL by ultrafiltration and stored at −80°C. Protein concentration was measured by UV spectrophotometry and purity was analyzed by sodium dodecyl sulfate-polyacrylamide gel electrophoresis.

1.1.3. Extraction of rMGST1

Enzyme preparation for rMGST1 protein was previously described.[8] The exact same protocol was followed. Livers from male Sprague□Dawley rats (180–240 g, starved overnight) were homogenized in 0.25 M sucrose by four up and down strokes in a Potter Elvehjem glass Teflon homogenizer to yield a 20% homogenate. All procedures were at 4°C. The homogenate was spun 15 min at 10,000 g and the supernatant a further 60 min at 100,000 g. Microsomes were resuspended and washed twice in 0.15 M Tris□hydrochloride (pH 8) by centrifugation at 100,000 g for 30 min. The resulting pellet was resuspended in 0.25 M sucrose at 10 mg/mL and activity determined on fresh material. Microsomes were resuspended in 10 mM potassium phosphate, pH 7, 20% glycerol, 1 mM GSH, 0.1 mM EDTA, and 1% Triton X□100 (buffer A). The rat liver microsome preparation was essentially that described by Ernster *et al.*[9] with the addition of an extra wash to diminish cytosolic GST contamination.

The resuspended microsomes (60 mL of buffer A) were solubilized by addition of buffer A containing 8% Triton X-100 (100 mL) for 15 min. Next, a purification step involving hydroxyapatite was performed in two alternate ways: (1) the solubilized material was loaded on a hydroxyapatite column 5 × 40 cm pre□equilibrated with buffer A and eluted with a linear gradient of 10 mM to 0.4 M potassium phosphate (pH 7) in 2 L at 1 mL/min. Fractions of 5 mL were collected, assayed for GST activity, and pooled (the enzyme elutes at approximately 0.2 M potassium phosphate); (2) the solubilized microsomes were added directly to hydroxyapatite

pre□equilibrated with buffer A (30 g hydroxyapatite slurry plus an additional 140 mL buffer A directly in 1 L centrifuge flasks) in batch and mixed for 20 min by occasional gentle swirling. The in batch and mixed for 20 min by occasional gentle swirling. The hydroxyapatite was pelleted by low speed centrifugation (a pulse of 3000 rpm was used throughout) and the supernatant discarded. The hydroxyapatite was first washed twice with 200 mL of buffer A, then with 200 mL buffer A containing 50 mM potassium phosphate (pH 7) and subsequently with 2–3x 100 mL washes of buffer A containing 200 mM potassium phosphate (pH 7) in which the enzyme activity elutes. The eluates from procedure 1 or 2 were combined and subjected to gel filtration on a G-25 (fine) column (10 × 80 cm) equilibrated with buffer A to reduce potassium phosphate concentration.

The G□25 pool was added to a CM Sepharose column (2.5 × 15 cm) rate with a gradient of 0–0.2 M potassium chloride in 200 mL buffer A where the enzyme elutes at approximately 0.1 M potassium chloride; 3 mL fractions were collected, assayed for activity, and pooled. For additional purification, this step was repeated but with all buffers and the enzyme pool adjusted to pH 8. Top fractions and side fractions were pooled separately to yield one highly concentrated (1–3 mg/mL) and one less concentrated pool. Protein was determined by the method of Peterson[10] including the precipitation step. This protein assay has been calibrated with amino acid analysis and found to yield the true protein content with bovine serum albumin as a standard. Purity was determined by SDS□PAGE according to Laemmli[11] but including the double concentration of Tris hydrochloride in the separation gel. More SDS (6%) is sometimes needed in the sample buffer to balance Triton X-100. The pools were frozen in portions of 1–5 mL under nitrogen and stored at −80°C.

2. Supporting Information: References

1. Geib T, Lento C, Wilson DJ, Sleno L. Liquid chromatography-tandem mass spectrometry analysis of acetaminophen covalent binding to glutathione S-transferases. *Front Chem*. 2019;7:558. doi:10.3389/fchem.2019.00558
2. Mukanganyama S, Widersten M, Naik YS, Mannervik B, Hasler JA. Inhibition of glutathione S-transferases by antimalarial drugs possible implications for circumventing anticancer drug resistance. *Int J Cancer*. 2002;97(5):700-705. doi:10.1002/ijc.10054
3. Groom H, Lee M, Patil P, Josephy PD. Inhibition of human glutathione transferases by dinitronaphthalene derivatives. *Arch Biochem Biophys*. 2014;555-556:71-76. doi:10.1016/j.abb.2014.06.002
4. Habig WH, Pabst MJ, Jakoby WB. Glutathione S-transferases. The first enzymatic step in mercapturic acid formation. *J Biol Chem*. 1974;249(22):7130-7139. http://www.jbc.org/content/249/22/7130.long.
5. Qazi SS, Osoria Pérez A, Sam M, Leslie EM. Glutathione transferase P1 interacts strongly with the inner leaflet of the plasma membrane. *Drug Metab Dispos*. 2011;39(7):1122-1126. doi:10.1124/dmd.111.039362.through
6. Loe DW, Almquist KC, Deeley RG, Cole SPC. Multidrug resistance protein (MRP)-mediated transport of leukotriene C4 and chemotherapeutic agents in membrane vesicles. Demonstration of glutathione-dependent vincristine transport. *J Biol Chem*. 1996;271(16):9675-9682. doi:10.1074/jbc.271.16.9675
7. Ahmad S, Niegowski D, Wetterholm A, Haeggström JZ, Morgenstern R, Rinaldo-Matthis A. Catalytic characterization of human microsomal glutathione S -transferase 2: identification of rate-limiting steps. *Biochemistry*. 2013;52(10):1755-1764.

doi:10.1021/bi3014104

8. Morgenstern R. Microsomal glutathione transferase 1. *Methods Enzymol*. 2005;401:136-146. doi:10.1016/S0076-6879(05)01008-6

9. Ernster L, Siekevitz P, Palade GE. Enzyme-Structure Relationships in the Endoplasmic Reticulum of Rat Liver. *J Cell Biochem*. 1962;15(3):541-562. doi:10.1083/jcb.15.3.541

10. Peterson GL. A simplification of the protein assay method of Lowry et al. which is more generally applicable. *Anal Biochem*. 1977;83(2):346-356. doi:10.1016/0003-2697(77)90043-4

11. Laemmli UK. Cleavage of structural proteins during the assembly of the head of bacteriophage T4. *Nature*. 1970;227(5259):680-685. doi:10.1038/227680a0

3. Supporting Information: Tables

3.1. Table S1

Table S1. List of MRM transitions for relative SO quantitation of proteins (quantitative transitions underlined)

Protein	Site	Peptide precursor ion	Fragment ion	Q1 [*m/z*]	Q3 [*m/z*]	Collision energy [V]
**Trypsin digestion**						
hGSTP1	C15	C*AALR^{2+}	y_3	341.7	359.2	18
			y_4	341.7	430.3	20
			b_3	341.7	395.1	18
			y_1	341.7	175.1	28
		C*(d_4)AALR^{2+}	y_3	343.7	359.2	18
			y_4	343.7	430.3	20
			b_3	343.7	399.2	18
			y_1	343.7	175.1	28
	C48	ASC*LYGQLPK^{2+}	y_6	614.8	705.4	28
			y_7	614.8	818.5	28
			y_8	614.8	1070.5	27
			y_5	614.8	542.3	27
		ASC*(d_4)LYGQLPK^{2+}	y_6	616.8	705.4	28
			y_7	616.8	818.5	28
			y_8	616.8	1074.6	27
			y_5	616.8	542.3	27
hMGST1	C50	VFANPEDC*VAFGK^{2+}	y_9	773.4	1114.5	30
			y_8	773.4	1017.4	30
			y_7	773.4	888.4	30
			b_4	773.4	432.2	30
		VFANPEDC*(d_4)VAFGK^{2+}	y_9	775.4	1118.5	30
			y_8	775.4	1021.5	30
			y_7	775.4	892.4	30
			b_4	775.4	432.2	30
rMGST1	C50	VFANPEDC*AGFGK^{2+}	y_9	752.3	1072.4	30
			y_{10}	752.3	1186.5	30

			y_8	752.3	975.4	30
			b_4	752.3	432.2	30
		VFANPEDC*(d_4)AGFGK^{2+}	y_9	754.3	1076.5	30
			y_{10}	754.3	1190.5	30
			y_8	754.3	979.4	30
			b_4	754.3	432.2	30
rCYP2C6	C372	FIDLIPTNLPHAVTC*DIK^{3+}	y_{13}^{2+}	720.4	779.4	30
			y_{14}^{2+}	720.4	835.9	30
			b_{16}^{2+}	720.4	950.5	30
			b_5	720.4	602.4	30
		FIDLIPTNLPHAVTC*(d_4)DIK^{3+}	y_{13}^{2+}	721.7	781.4	30
			y_{14}^{2+}	721.7	837.9	30
			b_{16}^{2+}	721.7	952.5	30
			b_5	721.7	602.4	30
**Pepsin digestion**						
hGSTM2	C115	AKLC*YDPDF^{2+}	b_5	610.8	728.3	20
			b_6	610.8	843.4	25
			b_8	610.8	1055.5	25
			y_3	610.8	378.2	25
		AKLC*(d_4)YDPDF^{2+}	b_5	612.8	732.4	20
			b_6	612.8	847.4	25
			b_8	612.8	1059.5	25
			y_3	612.8	378.2	25
	C174	ERNQVFEPSC*L^{2+}	b_6	735.8	774.4	30
			b_7	735.8	903.4	30
			b_9	735.8	1087.5	30
			y_4	735.8	568.2	30
		ERNQVFEPSC*(d_4)L^{2+}	b_6	737.9	774.4	30
			b_7	737.9	903.4	30
			b_9	737.9	1087.5	30
			y_4	737.9	572.3	30
hGSTP1	C170	IHEVLAPGC*L^{2+}	y_5	600.8	609.3	23
			b_6	600.8	663.4	23

			b_8	600.8	817.5	27
			y_4	600.8	538.2	23
		IHEVLAPGC*(d$_4$)L^{2+}	y_5	602.8	613.3	23
			b_6	602.8	663.4	23
			b_8	602.8	817.5	27
			y_4	602.8	542.3	23
hSA	C34	LQQC*PFEDHVKL^{3+}	y_8	535.9	984.4	16
			PFEDH$^+$	535.9	626.1	35
			$(y_{10}-H_2O)^{2+}$	535.9	673.9	22
		LQQC*(d$_4$)PFEDHVKL^{3+}	y_8	537.2	984.4	16
			PFEDH$^+$	537.2	626.3	35
			$(y_{10}-H_2O)^{2+}$	537.2	675.8	22

3.2. Table S2

Table S2. Measured NAPQI-protein SO (averaged, %CV of repeated injections, *n* = 3)

				1% isotope dilution		0.1% isotope dilution		0.01% isotope dilution	
**Incubation system**	**Protein**	**Site**	**MRM transition (peptide/fragment)**	**SO [%]**	**%CV**	**SO [%]**	**%CV**	**SO [%]**	**%CV**
hCYP3A4 Supersomes	hGSTP1	Cys15	C*AALR^{2+}/y_3	-	-	-	-	0.0001	45.8
		Cys48	ASC*LYGQLPK^{2+}/y_6	-	-	-	-	0.0005	19.6
		Cys170	IHEVLAPGC*L^{2+}/b_6	-	-	-	-	0.0025	3.1
hCYP3A4 Supersomes	His-hGSTP1	Cys15	C*AALR^{2+}/y_3	-	-	-	-	0.0004	8.6
		Cys48	ASC*LYGQLPK^{2+}/y_6	-	-	-	-	0.0048	25.6
		Cys170	IHEVLAPGC*L^{2+}/b_6	-	-	-	-	0.0089	0.5
hCYP3A4 Supersomes	hGSTM2	Cys115	AKLC*YDPDF^{2+}/b_6	-	-	0.0148	7.2	0.0082	12.6
		Cys174	ERNQVFEPSC*L^{2+}/b_7	-	-	0.0050	0.6	0.0046	2.3
hCYP3A4 Supersomes	hMGST1	Cys50	VFANPEDC*VAFGK^{2+}/y_9	-	-	0.0276	2.6	0.0248	4.6
hCYP3A4 Supersomes	rMGST1	Cys50	VFANPEDC*AGFGK^{2+}/y_9	-	-	0.0016	8.4	0.0017	2.8
HLM	hMGST1	Cys50	VFANPEDC*VAFGK^{2+}/y_9	1.421	2.9	0.612	2.8	-	-
RLM	rMGST1	Cys50	VFANPEDC*AGFGK^{2+}/y_9	2.831	1.9	2.548	1.4	-	-
	rCYP2C6	Cys372	FIDLIPTNLPHAVTC*DIK^{3+}/y_{13}^{2+}	0.621	3.1	0.508	11.5	-	-
